# A Case Report of Nebulized Tranexamic Acid for Post-tonsillectomy Hemorrhage in an Adult

**DOI:** 10.5811/cpcem.2020.6.47676

**Published:** 2020-07-14

**Authors:** Michael Poppe, Felipe Grimaldo

**Affiliations:** Naval Medical Center San Diego, Department of Emergency Medicine, San Diego, California

**Keywords:** Tranexamic acid, TXA, post-tonsillectomy hemorrhage

## Abstract

**Introduction:**

Post-tonsillectomy hemorrhage is a potentially life-threatening, postoperative complication that is commonly encountered in the emergency department (ED).

**Case Report:**

Herein, we describe the case of a 22-year-old male who presented to the ED with an active post-tonsillectomy hemorrhage. He rapidly became hypotensive and experienced an episode of syncope. Immediate interventions included intravenous fluids, emergency release blood and nebulized tranexamic acid (TXA). After completion of the nebulized TXA, the patient’s bleeding was controlled.

**Conclusion:**

To our knowledge, this is the first case in the emergency medicine literature that describes the use of nebulized TXA in an adult to achieve hemostasis in post-tonsillectomy hemorrhage.

## INTRODUCTION

Postoperative hemorrhage is the leading cause of death associated with tonsillectomy, most commonly occurring on postoperative days five to seven.^1^ It is often a therapeutic challenge for emergency physicians, many times requiring management without surgical assistance. The American Academy of Otolaryngology-Head and Neck Surgery Foundation guidelines offer little assistance, as there is no report on the best way to control post-tonsillectomy hemorrhage. Management strategies in the adult population have not been extensively studied.

Management strategies in the pediatric population include intravenous (IV) fluids, direct pressure, clot suction, silver nitrate, vasoconstrictor-soaked pledgets, epinephrine injections, topical epinephrine, thrombin powder, and labs.^2^ Tranexamic acid (TXA) is an antifibrinolytic, which has been studied extensively in many different settings for its procoagulant properties. There is a limited body of published literature describing inhaled TXA use, as well as oral TXA, for post-tonsillectomy hemorrhage in the pediatric population.^3^ Here, we present the first case report of inhaled TXA use in the setting of adult post-tonsillectomy hemorrhage.

## CASE REPORT

A 22-year-old male presented to the emergency department (ED) with active post-tonsillectomy hemorrhage. He was post-operative day five from tonsillectomy performed for recurrent tonsillitis. Approximately one hour prior to arrival, he had been eating pizza rolls when he felt a “scratch in his throat.” He began bleeding profusely, unable to speak more than one to two words at a time before his mouth would fill with blood. By the time he arrived to the ED, he had filled an emesis basin with approximately 500 milliliters (mL) of blood. He was found to have a tachycardia of 104 beats per minute (bpm) with a blood pressure of 131/94 millimeters of mercury (mmHg). Due to the large volume of blood in the basin, continued bleeding, and tachycardia, one unit of uncrossmatched packed red blood cells (PRBC) was transfused. Both TXA (1000 milligrams [mg] per 10 mL) five mL and normal saline five mL were added to a nebulizer and administered to the patient.

Approximately 20 minutes into his ED evaluation, the patient became pale and diaphoretic. His heart rate increased to 122 bpm and blood pressure significantly decreased to 75/40 mmHg. Massive transfusion protocol was initiated, and the patient required a total of two units of PRBCs before his blood pressure and heart rate normalized. Upon completing the nebulizer treatment, the patient’s rate of bleeding slowed and he could then speak full sentences. Examination of the oropharynx revealed a left fossa with postsurgical exudate without any active bleeding and a right fossa filled with clot and a steady, small-volume flow of bright red blood. Otolaryngology arrived at bedside and performed bedside coagulation cautery, after which bleeding was completely controlled. He was admitted to the hospital for further observation and discharged the following day.

## DISCUSSION

TXA is an analog of the amino acid lysine. It functions as an antifibrinolytic by binding to plasminogen, inhibiting its transformation into plasmin, which in turn results in decreased fibrin breakdown. TXA has been studied for a variety of bleeding conditions in the adult and pediatric populations. Initially designed to assist in postpartum hemorrhage, its uses have been broadened in the interim.^4^ In the trauma setting it has also been shown to have a mortality benefit when given in the first three hours from injury.^5^

While generally considered a benign medication, it is not without risks. IV administration has been associated with increased risk of pulmonary embolism and deep venous thrombosis. This risk seems to increase if given after the first three hours of bleeding onset.^6^ That being said, these effects have not been studied in patients given TXA via a nebulized route. It has been studied in the setting of intracranial hemorrhage as well, with some evidence showing no statistical mortality benefit with increased risk of thromboembolic events.^7^ Areas of ongoing study where the inhaled route has been evaluated include diffuse alveolar hemorrhage (DAH) and hemoptysis.^8^–^12^ In diffuse alveolar hemorrhage models, doses of 250 milligrams (mg) for less than 25 kilogram (kg) patients and 500 mg for greater than 25kg patients were able to control intractable DAH in 10 out of 18 patients.^12^ As for hemoptysis patients, nebulized TXA has been shown to decrease the rate of bleed, decrease length of disease course, and decrease the need for invasive procedures when compared to placebo.^13^

In the pediatric population, there are case reports of nebulized TXA for post-tonsillectomy bleeding that have demonstrated feasibility in terms of ease of access to materials including TXA and nebulizers, as well as patient compliance and potential positive benefit.^14^ Cases described, as mentioned above, typically use doses of 250–500 mg nebulizer treatment, and most often efficacy is based on cessation of bleeding. Many of these involve co-treatment with nebulized epinephrine as well, which makes drawing conclusions as to efficacy from TXA alone more difficult. In this case, TXA and blood transfusion were the sole interventions, which resulted in clinical improvement and stabilization prior to the arrival of the otolaryngologist.

**CPC-EM Capsule**

What do we already know about this clinical entity?

Post-tonsillectomy hemorrhage is a common, potentially life-threatening complication seen frequently in the emergency department.

What makes this presentation of disease reportable?

There is weak evidence supporting the use of tranexamic acid (TXA) for pediatric post-tonsillectomy hemorrhage, but no evidence as to its effect on adults.

What is the major learning point?

TXA appears safe and with potential benefit in the setting of post-tonsillectomy hemorrhage in an adult.

How might this improve emergency medicine practice?

This novel treatment modality for post-tonsillectomy hemorrhage in an adult adds a tool to the emergency provider’s arsenal.

While a single case is not sufficient for determining causative relationships, the pharmacology of TXA and time course do support its efficacy in this case. The estimated onset of effect for TXA is one to two minutes, with an expected clinical effect to occur at 10–30 minutes.^15^ This seems consistent with the timing of this patient’s clinical course, as bleeding had slowed down significantly to the point that he could speak and be fully examined approximately 25 minutes after administration. Some concern may be raised as to whether therapeutic concentrations may be obtained via a nebulized route. In vitro studies have shown a plasma concentration of 16 micrograms/mL to be the threshold for ceasing fibrinolysis. This patient received a total of 1000 mg of TXA. Even allowing for incomplete dosing due to frequent spitting, the patient would need to have absorbed a fraction of the initial 100 mg/mL dose to achieve effective concentration. In addition, it has been hypothesized that topical oral administration may be especially effective, given there is a relatively high concentration of plasminogen and low concentration of plasmin inhibitors in the oral cavity.^9^ In pediatric studies of DAH, TXA has not been linked to any adverse effects (seizures, thromboembolic events, worsening of gas exchange).^12^ Thus there is minimal risk in attempting treatment. This is in contrast to IV or oral TXA, which has at times been linked to increased risk of thromboembolism, renal injury, or hypotension if administered too quickly although the evidence remains inconclusive.^9^

## CONCLUSION

Massive post-tonsillectomy bleeds can leave physicians feeling helpless due to limited treatment modalities, the potential for extremely difficult-to-control airway scenarios, and possibly prolonged duration of care before operative intervention becomes available. In the pediatric population both inhaled epinephrine and inhaled TXA have proven efficacious and safe. A well-documented risk factor for post-tonsillectomy bleeding is increased age, putting adults at potentially higher risk. In adult patients, this case demonstrated inhaled TXA to be a safe and effective management technique in addition to resuscitative care while waiting for surgical intervention.
